# Supplementary material for: The SUMO–NIP45 pathway processes toxic DNA catenanes to prevent mitotic failure
Source: Nat Struct Mol Biol. 2023 Jul 20;30(9):1303–13. doi: 10.1038/s41594-023-01045-0 (PMC10497417; doi:10.1038/s41594-023-01045-0)
Supplement: Supplementary file 1 — Supplementary Tables 1–3, legend to Video 1, Methods and References. [file 41594_2023_1045_MOESM1_ESM.pdf]

nature structural & molecular biology

Article https://doi.org/10.1038/s41594-023-01045-0

The SUMO–NIP45 pathway processes toxic DNA catenanes to prevent mitotic failure

In the format provided by the authors and unedited

Contents

Supplementary Tables 1-3 with legends

Legend to Supplementary Video 1

Supplementary Methods

References for Supplementary Information

Supplementary Tables

Primer	Sequence (5' - 3')
Fw_EGFP-N_HindIII	CCCGGGAAGCTTCCACCATGGTGAGCAAGGGCGAGGAGC
Rw_EGFP-N_HindIII	CCCGGGAAGCTTCTTGTACAGCTCGTCCATGC
Fw_NIP45_KpnI	CCCGGGGGTACCATGGCGGAGCCTGTGGGGAAGC
Rv_NIP45_NotI	CCCGGGGCGGCCGCTCAGCCCCAGACCTCAATGAGG
Fw_mCherry_AflII	CCCGGGCTTAAGCCACCATGGTGAGCAAGG
Rv_mCherry_KpnI	CCCGGGGGTACCCTTGTACAGCTCGTCCATGC
Fw_NIP45_SLD1	ACAAGTTCTCCAGAGGCCACAG
Rv_NIP45_SLD1	CTCTGGGAGGGTGGGCCC
Rv_NIP45_SLD2_NotI	CCCGGGGCGGCCGCTCACTCTGTGGCCTCTGGAGAAC
Fw_NIP45_208-419_KpnI	CCCGGGGGTACCGGCGAAGGGCAAGGGCAAGGGCAAGGGCCGGGCCGCGGC AAAAGCAGAACGCATACTCG
Fw_NIP45_261-419_KpnI	CCCGGGGGTACCGGCGAAGGGCAAGGGCAAGGGCAAGGGCCGGGCCGCGGC ACCCCCCGACTCTTCCCACTC
Fw_NIP45_D394R	GCTCTCCTTCTTCTTTCGTGGGACAAAGCTTTCAGG
Rv_NIP45_D394R	CCTGAAAGCTTTGTCCCACGAAAGAAGAAGGAGAGC
pLCKO2_forward	GAGGGCCTATTTCCCATGATTC
pLCKO2_reverse	CAAACCCAGGGCTGCCTTGGAA
LCV2_forward	GAGGGCCTATTTCCCATGATTC
LCV2_reverse	GTTGCGAAAAAGAACGTTCACGG

Supplementary Table 1. List of all primers used in this study.

Antibody	Type	Source	Identifier	Dilution
GAPDH	rabbit polyclonal	Santa Cruz	sc-25778	1:1,000
BLM	rabbit polyclonal	Bethyl Laboratories	A300-110A	1:1,000
Actin	mouse monoclonal	Merck	MAB1501	1:20,000
RMI2	rabbit polyclonal	Abcam	ab122685	1:750
FKBP8	rabbit monoclonal	Abcam	ab129113	1:1,000
P300	rabbit polyclonal	Abcam	ab10485	1:5,000
GFP	rabbit polyclonal	Chromotek	PABG1	1:2,000
GFP	mouse monoclonal	Merck	11814460001	1:1,000
SUMO2/3	rabbit polyclonal	Abcam	ab3742	1:1,000
Histone H3	rabbit polyclonal	Abcam	ab1791	1:50,000
MUS81	mouse monoclonal	Santa Cruz	sc-53382	1:250
ATM phospho-S1981	rabbit monoclonal	Abcam	ab81292	1:1,000
ATM	mouse monoclonal	Santa Cruz	sc-135663	1:100
KAP1 phospho-S824	rabbit polyclonal	Bethyl Laboratories	A300-767A	1:1,000
KAP1	rabbit polyclonal	Bethyl Laboratories	A300-274A	1:1,000
Chk2 phospho-T68	rabbit polyclonal	Cell Signaling Technologies	2661	1:500
Chk2	mouse monoclonal	Invitrogen	MA5-31595	1:500
Chk2	rabbit monoclonal	Abcam	ab109413	1:1,000
H2AX phospho-S139	rabbit polyclonal	Cell Signaling Technologies	2577	1:500
H2AX phospho-S139	mouse monoclonal	Merck	05-636	1:500
Chk1 phospho-S345	rabbit polyclonal	Cell Signaling Technologies	2348	1:1,000
Vinculin	mouse monoclonal	Merck	V9131	1:10,000
EME1	mouse monoclonal	Santa Cruz	sc-53275	1:100
TOP2A	mouse monoclonal	Santa Cruz	sc-166934	1:500
UBC9	goat polyclonal	Abcam	ab21193	1:500
53BP1	rabbit polyclonal	Novus Biologicals	NB100-304	1:500
NBS1	rabbit polyclonal	Santa Cruz	sc-11431	1:1000
SLX4	rabbit polyclonal	Abcam	ab100997	1:1,000
MPM2 phospho-SP/TP Cy5	mouse monoclonal	Merck	16-220	1:500

Supplementary Table 2. List of all commercially available antibodies used in this study.

siRNA	Vendor	Type	Sequence (5'-3')	Conc. (nM)
siCTRL	Thermo Fisher	Silencer Select	Ambion Silencer Negative Control # 1; sequence undisclosed	10
siRMI1	Thermo Fisher	Silencer Select	GAGUUGUUCUUUAAGAUCA[dT][dT]	10
siRMI2	Thermo Fisher	Silencer Select	GAAACUAUUUAGAAGCUUA[dT][dT]	10
siCRAMP1L	Thermo Fisher	Silencer Select	CUGUCUAACUCUUCCGUAA[dT][dT]	10
siFKBP8	Thermo Fisher	Silencer Select	CUCCUGCAGUUGAAGGUGA[dT][dT]	10
siP300	Thermo Fisher	Silencer Select	GCCUGGUUAUAUAACCGGA[dT][dT]	10
siNIP45#1	Thermo Fisher	Silencer Select	GCCUUCGCCUUAUCCCAGA[dT][dT]	10
siNIP45#2	Thermo Fisher	Silencer Select	GAGGACUAAGGAUAAAGAA[dT][dT]	10
siBLM#1	Sigma	Custom siRNA	GCAACUAGAACGUCACUCA[dT][dT]	50
siBLM#2	Sigma	Custom siRNA	GGAUGACUCAGAAUGGUUA[dT][dT]	50
siBLM#3	Sigma	Custom siRNA	GAUCAAUGCUGCACUGCUU[dT][dT]	50
siBLM#4	Sigma	Custom siRNA	CUAAAUCUGUGGAGGGUUA[dT][dT]	50
siPICH[1]	Sigma	Custom siRNA	AGUAGGUGGUGUCGGUUUA[dT][dT]	50
siSLX4#1[2]	Sigma	Custom siRNA	GAGAAGAACCCUAAUGAAA[dT][dT]	50
siSLX4#2[2]	Sigma	Custom siRNA	GCACAAGGGCCCAGAACAA[dT][dT]	50
siSAE1	Sigma	Custom siRNA	AGAAGAAACCAGAGUCAUU[dT][dT]	50
siEME1[3]	Sigma	Custom siRNA	GCUAAGCAGUGAAAGUGAA[dT][dT]	50
siNBS1#1	Sigma	Custom siRNA	ACAUGGGAUUUGAGUGAAA[dT][dT]	50
siNBS1#2	Sigma	Custom siRNA	UGACACAGAAUCAGAGCAA[dT][dT]	50
siNBS1#3	Sigma	Custom siRNA	GGAUAUGCUCCAAAGGCAA[dT][dT]	50

Supplementary Table 3. List of all siRNAs used in this study.

Legend to Supplementary Video 1

Supplementary Video 1.
Cytokinesis failure and binucleation in SUMOi-treated NIP45-KO cells
Representative example of abortive cytokinesis and binucleation in SUMOi-treated NIP45-KO cells in **Figure 3B**.

Supplementary Methods

GFP-trap pull-downs for MS analysis

Quadruplicates of U2OS Flp-In T-REx NIP45-KO/GFP-NIP45 WT and SLD2* cell lines were induced with 0.1 µM doxycycline for 24 h and cell pellets collected and lysed for 15 min in ice-cold low-salt buffer containing 1 mM NaF, 10 mM N-Ethylmaleimide, 10 mM β-glycerophosphate, 0.1 mM vanadate and complete EDTA-free protease inhibitor cocktail (Roche). Cell lysates were cleared by centrifugation at 16,100×g at 4 °C. Protein concentration was determined using the Pierce BCA protein assay kit (Thermo Fisher Scientific) and equalized with lysis buffer before addition to 30 µL GFP-trap bead/slurry pre-washed twice in lysis buffer. Following incubation at 4 °C for 45 min the beads were washed three times in lysis buffer. Digestion of proteins was performed on-beads, using 250 ng trypsin, with 30 min pre-incubation on ice and 3 h incubation with shaking at 37 °C. Digests were cleared by centrifugation through 0.45 µm spin filters, after which peptides were purified on StageTips at high pH [4]. C18 StageTips were prepared in-house, by layering four plugs of C18 material (Sigma-Aldrich, Empore SPE Disks, C18, 47 mm) per StageTip. Activation of StageTips was performed with 100 µL 100% methanol, followed by equilibration using 100 µL 80% acetonitrile (ACN) in 200 mM ammonium hydroxide, and two washes with 100 µL 50 mM ammonium hydroxide. Samples were basified to pH >10 by addition of one tenth volume of 200 mM ammonium hydroxide, after which they were loaded on StageTips. Subsequently, StageTips were washed twice using 100 µL 50 mM ammonium hydroxide, after which peptides were eluted using 80 µL 25% ACN in 50 mM ammonium

hydroxide. All fractions were dried to completion using a SpeedVac at 60 °C. Dried peptides were dissolved in 10 μL 0.1% formic acid (FA) and stored at −20 °C until analysis using mass spectrometry (MS).

Enrichment of SUMOylated proteins for MS and immunoblotting analysis
Quadruplicates of HeLa/His_{10}-SUMO2 cells and the parental HeLa cell line were treated with siRNAs for 72 h prior to incubation with or without 2 μM ICRF-193 for 2 h. Purification of proteins modified by His_{10}-SUMO2 was performed essentially as described previously [5]. Briefly, cells were washed twice with ice-cold PBS, scraped, and lysed in 10 pellet volumes of Guanidine Lysis Buffer (6 M guanidine-HCl; 93.2 mM Na2HPO4; 6.8 mM NaH_2PO_4; 10 mM Tris-HCl, pH 8.0). Lysates were homogenized using sonication (2–4 bursts of 5 s sonication at ~30 W), supplemented with 50 mM imidazole and 5 mM 2-mercaptoethanol, and 50 μl of pre-equilibrated Ni-NTA agarose beads were added per sample. Beads were incubated overnight at 4 °C in a rotator-mixer, after which they were washed in order with Wash Buffer 1 (6 M guanidine-HCl; 93.2 mM Na_2HPO_4; 6.8 mM NaH_2PO_4; 10 mM Tris-HCl, pH 8.0; 0.1 % Triton X-100; 10 mM imidazole; 5 mM 2-mercaptoethanol), Wash Buffer 2 (8 M urea; 93.2 mM Na_2HPO_4; 6.8 mM NaH_2PO_4; 10 mM Tris-HCl, pH 8.0; 0.1 % Triton X-100; 10 mM imidazole; 5 mM 2-mercaptoethanol), Wash Buffer 3 (8 M urea; 21.6 mM Na_2HPO_4; 78.4 mM NaH_2PO_4; 10 mM Tris-HCl, pH 6.3; 10 mM imidazole; 5 mM 2-mercaptoethanol), and twice with Wash Buffer 4 (8 M urea; 21.6 mM Na_2HPO_4; 78.4 mM NaH_2PO_4; 10 mM Tris-HCl, pH 6.3; 5 mM 2-mercaptoethanol). Beads were eluted twice using one bead volume of Elution Buffer (7 M urea; 58 mM Na_2HPO_4; 42 mM NaH_2PO_4; 10 mM Tris-HCl, pH 7.0; 500 mM imidazole), after which elutions were cleared through 0.45 μm spin filters. Samples were concentrated using 100K MWCO spin filters (Sartorius Stedim), discarding the flowthrough (which only contains unconjugated SUMO). Samples were washed twice on the 100K MWCO spin filters using 250 μl of Urea Buffer (8 M urea; 93.2 mM Na_2HPO_4; 6.8 mM NaH_2PO_4; 10 mM Tris-HCl, pH 8.0), after which the volume was increased to 50 μl and ammonium bicarbonate added to a final concentration of 50 mM. Reduction and alkylation of peptides was performed by simultaneous addition of chloroacetamide and tris(2-carboxyethyl)phosphine to final concentrations of 5 mM, and afterwards digested with 1:50 (w/w) Lys-C for 3 h at room temperature, diluted to 2 M urea using 3 volumes of 50 mM ammonium bicarbonate, and digested overnight with 1:50 (w/w) trypsin. Following digestion, StageTip purification of peptides was performed at high pH, with a single-shot elution, as described above, after which samples were stored at −20°C until MS analysis.

MS analysis
All MS samples were analyzed on an EASY-nLC 1200 system (Thermo) coupled to an Orbitrap Exploris 480 mass spectrometer (Thermo). Samples were analyzed on 20 cm long analytical columns, with an internal diameter of 75 μm, and packed in-house using ReproSil-Pur 120 C18-AQ 1.9 µm beads (Dr. Maisch). The analytical column was heated to 40 °C, and elution of peptides from the column was achieved by application of gradients with stationary phase Buffer A (0.1% FA) and increasing amounts of mobile phase Buffer B (80% ACN in 0.1% FA). The primary analytical gradient ranged from 7 %B to 33 %B over 75 min, followed by a tail-end increase to 48 %B over 11 min to ensure full peptide elution, followed by a washing block of 14 min. Ionization was achieved using a NanoSpray Flex NG ion source (Thermo), with spray voltage set at 2 kV, ion transfer tube temperature to 275 °C, and RF funnel level to 40%. Full scan range was set to 300-1,300 *m/z*, MS1 resolution to 120,000, MS1 AGC target to “200” (2,000,000 charges), and MS1 maximum injection time to “Auto”. Precursors with charges 2-6 were selected for fragmentation using an isolation width of 1.3

m/z and fragmented using higher-energy collision disassociation (HCD) with normalized collision energy of 25. Monoisotopic Precursor Selection (MIPS) was enabled in “Peptide” mode. Precursors were prevented from being repeatedly sequenced by setting expected peak width to 50 s, and setting dynamic exclusion duration to 100 s, with an exclusion mass tolerance of 15 ppm, exclusion of isotopes, and exclusion of alternate charge states for the same precursor. MS/MS resolution was set to 30,000, MS/MS AGC target to “200” (200,000 charges), MS/MS intensity threshold to 360,000, MS/MS maximum injection time to “Auto”, and TopN to 12.

MS raw data analysis
MS proteomics RAW data are available at the ProteomeXchange Consortium database via the Proteomics Identifications (PRIDE) partner repository [6], under dataset ID PXD033739 (reviewer account: reviewer_pxd033739@ebi.ac.uk and password: MkZTXKw6). All RAW files were analyzed using MaxQuant software (version 1.5.3.30) [7,8]. RAW files corresponding to the GFP-trap and SUMOylome experiments were analyzed separately. Default MaxQuant settings were used, with exceptions outlined below. For generation of the theoretical spectral library, the HUMAN.FASTA database was extracted from UniProt on 24 May, 2019. Protein N-terminal acetylation and methionine oxidation were included as potential variable modifications (default), with a maximum allowance of 3 variable modifications per peptide. First search mass tolerance was set to 10 ppm, and maximum charge state of considered precursors to 6. Label-free quantification (LFQ) was enabled, “Fast LFQ” was disabled, and “LFQ min. ratio count” was increased to 3. Second peptide search was enabled (default) and matching between runs was enabled with a match time window of 1 min and an alignment time window of 20 min. Data was filtered by posterior error probability to achieve a false discovery rate of <1% (default), at both the peptide-spectrum match and the protein assignment levels.

Statistical analysis and visualization of MS data
Processing of the text file output by MaxQuant was performed using Perseus software [9]. Reverse-database hits and potential contaminant proteins were removed. LFQ intensities were log2 transformed for further analyses. Proteins not detected in 4 out of 4 biological replicates in at least one experimental condition were removed. Scatter plot analysis, principal component analysis, Z-scoring, profile clustering, and two-sample testing for the generation of volcano plots, were all carried out in Perseus using default settings. For two-sample testing on the GFP IP samples, an s0 of 0.5 was used for comparing mutant to wt IPs, and an s0 of 0.1 was used for comparing ICRF-193 to mock treatment. For the SUMOylome experiments, the s0 value was set to 1 for two-sample testing between the parental control and SUMO-enriched samples, and to 0.5 for testing between the differentially treated SUMO samples. For detection of significant differences between differentially treated SUMO samples, proteins were only considered if they were significantly enriched over the parental control in any of the SUMO-enriched conditions. Differences observed through two-sample testing were considered significant at a q-value of <0.05, representing the corrected p-value after application of permutation-based FDR control.

Supplementary References

1. Hubner, N.C., Wang, L.H., Kaulich, M., Descombes, P., Poser, I., and Nigg, E.A. (2010). Re-examination of siRNA specificity questions role of PICH and Tao1 in the spindle checkpoint and identifies Mad2 as a sensitive target for small RNAs. Chromosoma *119*, 149-165. 10.1007/s00412-009-0244-2.

2. Wilson, J.S., Tejera, A.M., Castor, D., Toth, R., Blasco, M.A., and Rouse, J. (2013). Localization-dependent and -independent roles of SLX4 in regulating telomeres. Cell reports *4*, 853-860. 10.1016/j.celrep.2013.07.033.

3. Naim, V., Wilhelm, T., Debatisse, M., and Rosselli, F. (2013). ERCC1 and MUS81-EME1 promote sister chromatid separation by processing late replication intermediates at common fragile sites during mitosis. Nat Cell Biol *15*, 1008-1015. 10.1038/ncb2793.

4. Hendriks, I.A., Lyon, D., Su, D., Skotte, N.H., Daniel, J.A., Jensen, L.J., and Nielsen, M.L. (2018). Site-specific characterization of endogenous SUMOylation across species and organs. Nat Commun *9*, 2456. 10.1038/s41467-018-04957-4.

5. Hendriks, I.A., and Vertegaal, A.C. (2016). Label-Free Identification and Quantification of SUMO Target Proteins. Methods Mol Biol *1475*, 171-193. 10.1007/978-1-4939-6358-4_13.

6. Vizcaino, J.A., Deutsch, E.W., Wang, R., Csordas, A., Reisinger, F., Rios, D., Dianes, J.A., Sun, Z., Farrah, T., Bandeira, N., et al. (2014). ProteomeXchange provides globally coordinated proteomics data submission and dissemination. Nat Biotechnol *32*, 223-226. 10.1038/nbt.2839.

7. Cox, J., and Mann, M. (2008). MaxQuant enables high peptide identification rates, individualized p.p.b.-range mass accuracies and proteome-wide protein quantification. Nat Biotechnol *26*, 1367-1372. nbt.1511 [pii] 10.1038/nbt.1511.

8. Cox, J., Neuhauser, N., Michalski, A., Scheltema, R.A., Olsen, J.V., and Mann, M. (2011). Andromeda: a peptide search engine integrated into the MaxQuant environment. J Proteome Res *10*, 1794-1805. 10.1021/pr101065j.

9. Tyanova, S., Temu, T., Sinitcyn, P., Carlson, A., Hein, M.Y., Geiger, T., Mann, M., and Cox, J. (2016). The Perseus computational platform for comprehensive analysis of (prote)omics data. Nat Methods *13*, 731-740. 10.1038/nmeth.3901.
