# Supplementary material for: The SUMO–NIP45 pathway processes toxic DNA catenanes to prevent mitotic failure
Source: Nat Struct Mol Biol. 2023 Jul 20;30(9):1303–13. doi: 10.1038/s41594-023-01045-0 (PMC10497417; doi:10.1038/s41594-023-01045-0)
Supplement: Supplementary file 2 — Reporting Summary [file 41594_2023_1045_MOESM2_ESM.pdf]

nature portfolio

Corresponding author(s): Niels Mailand

Last updated by author(s): Feb 14, 2023

Reporting Summary

Nature Portfolio wishes to improve the reproducibility of the work that we publish. This form provides structure for consistency and transparency in reporting. For further information on Nature Portfolio policies, see our Editorial Policies and the Editorial Policy Checklist.

Statistics

For all statistical analyses, confirm that the following items are present in the figure legend, table legend, main text, or Methods section.

n/a	Confirmed	
☐	☒	The exact sample size (*n*) for each experimental group/condition, given as a discrete number and unit of measurement
☐	☒	A statement on whether measurements were taken from distinct samples or whether the same sample was measured repeatedly
☐	☒	The statistical test(s) used AND whether they are one- or two-sided<br>*Only common tests should be described solely by name; describe more complex techniques in the Methods section.*
☒	☐	A description of all covariates tested
☐	☒	A description of any assumptions or corrections, such as tests of normality and adjustment for multiple comparisons
☐	☒	A full description of the statistical parameters including central tendency (e.g. means) or other basic estimates (e.g. regression coefficient) AND variation (e.g. standard deviation) or associated estimates of uncertainty (e.g. confidence intervals)
☐	☒	For null hypothesis testing, the test statistic (e.g. *F*, *t*, *r*) with confidence intervals, effect sizes, degrees of freedom and *P* value noted<br>*Give P values as exact values whenever suitable.*
☒	☐	For Bayesian analysis, information on the choice of priors and Markov chain Monte Carlo settings
☒	☐	For hierarchical and complex designs, identification of the appropriate level for tests and full reporting of outcomes
☒	☐	Estimates of effect sizes (e.g. Cohen's *d*, Pearson's *r*), indicating how they were calculated

Our web collection on statistics for biologists contains articles on many of the points above.

Software and code

Policy information about availability of computer code

Data collection	SoftWoRx (version 7.0.0; GE Healthcare), bcl2fastq (version 2.19.1), cutadapt (version 1.18), ImageStudio (version 3.1.4; LI-COR), ImageQuant LAS4000 software (version 1.2; GE Healthcare), Incucyte S3 Live-Cell Analysis System (version 2021A), BD FACS Diva software (version 9.0)
Data analysis	ScanR analysis software (version 2.8.1; Olympus), Spotfire (version 10.5.0; Tibco), SoftWoRx software (version 7.0.0; GE Healthcare), TriTek CometScore (version 2.0), GraphPad Prism (version 9.3.0), MaxQuant (version 1.5.3.30), Perseus (Tyanova et al., Nat Methods 13: 731-740 (2016)), R (https://www.r-project.org/), MAGeCK (version 0.5.8), drugZ (Colic et al., Genome Med 11: 52 (2019), BAGEL (Hart and Moffat, BMC Bioinformatics 17: 164 (2016)), ImageStudio (version 3.1.4; LI-COR), ImageQuant LAS4000 software (version 1.2; GE Healthcare), FLUOstar Omega MARS (version V4.01; BMG Labtech), Incucyte S3 Live-Cell Analysis System (version 2021A), FCS Express (version 7; DeNovo Software), Fiji (version 2.3.0/1.53q)

For manuscripts utilizing custom algorithms or software that are central to the research but not yet described in published literature, software must be made available to editors and reviewers. We strongly encourage code deposition in a community repository (e.g. GitHub). See the Nature Portfolio guidelines for submitting code & software for further information.

Data

Policy information about availability of data

All manuscripts must include a data availability statement. This statement should provide the following information, where applicable:

- Accession codes, unique identifiers, or web links for publicly available datasets
- A description of any restrictions on data availability
- For clinical datasets or third party data, please ensure that the statement adheres to our policy

The CRISPR screen data sets (Supplementary Data 1-5) are provided with this study. The mass spectrometry proteomics data (Supplementary Data 6-7) have been deposited to the ProteomeXchange Consortium 67 via the Proteomics Identifications (PRIDE) partner repository (http://www.ebi.ac.uk/pride) under dataset ID PXD033739 (reviewer account: reviewer_pxd033739@ebi.ac.uk and password: MkZTXKw6). sgRNA sequences in the TKOv3 library are available on addgene (). All other data supporting the findings of this study are available within the article and supplementary information.

Human research participants

Policy information about studies involving human research participants and Sex and Gender in Research.

Reporting on sex and gender	N/A
Population characteristics	N/A
Recruitment	N/A
Ethics oversight	N/A

Note that full information on the approval of the study protocol must also be provided in the manuscript.

Field-specific reporting

Please select the one below that is the best fit for your research. If you are not sure, read the appropriate sections before making your selection.

☒ Life sciences ☐ Behavioural & social sciences ☐ Ecological, evolutionary & environmental sciences

For a reference copy of the document with all sections, see nature.com/documents/nr-reporting-summary-flat.pdf

Life sciences study design

All studies must disclose on these points even when the disclosure is negative.

Sample size	Data were obtained according to the field's best practice. No statistical method was used to predetermine sample size. For CRISPR screens, sample sizes were designed based on publications using the same cell lines and CRISPR libraries (Hart et al., G3 7: 2719-2727 (2017); Olivieri et al., Cell 182: 481-496.e21 (2020)). Sample size for each experiment is indicated either in figure legends or methods.
Data exclusions	No data were excluded from the analyses.
Replication	All experimental findings shown in this study were independently replicated at least twice with similar outcome. Information about replication is provided in the figure legends.
Randomization	The samples were not randomized. Randomization is generally not relevant for this study since we are working with cell populations and not test subjects.
Blinding	The investigators were not blinded to group allocation during data collection and analysis. For most experiments, data analysis was performed computationally with fixed parameters across samples. For practical reasons, investigators were not blinded when scoring number of UFBs per cell but great measure was taken to avoid bias.

Reporting for specific materials, systems and methods

We require information from authors about some types of materials, experimental systems and methods used in many studies. Here, indicate whether each material, system or method listed is relevant to your study. If you are not sure if a list item applies to your research, read the appropriate section before selecting a response.

Materials & experimental systems

n/a	Involved in the study
☐	☒ Antibodies
☐	☒ Eukaryotic cell lines
☒	☐ Palaeontology and archaeology
☒	☐ Animals and other organisms
☒	☐ Clinical data
☒	☐ Dual use research of concern

Methods

n/a	Involved in the study
☒	☐ ChIP-seq
☐	☒ Flow cytometry
☒	☐ MRI-based neuroimaging

Antibodies

Antibodies used

The following commercially available antibodies were used: GAPDH (rabbit polyclonal, sc-25778, Santa Cruz; WB: 1:1,000), BLM (rabbit polyclonal, A300-110A, Bethyl Laboratories; WB: 1:1,000), Actin (mouse monoclonal clone C4, MAB1501, Merck; WB: 1:20,000), RMI2 (rabbit polyclonal, ab122685, Abcam; WB: 1:750), FKBP8 (rabbit monoclonal clone EPR7441(2), ab129113, Abcam; WB: 1:1,000), P300 (rabbit polyclonal, ab10485, Abcam; WB: 1:5,000), GFP (rabbit polyclonal, PABG1, Chromotek; WB: 1:2,000; IF: 1:2,000; mouse monoclonal clone 13.1, 11814460001, Merck; WB: 1:1,000), SUMO2/3 (rabbit polyclonal, ab3742, Abcam; WB: 1:1,000), Histone H3 (rabbit polyclonal, ab1791, Abcam; WB: 1:50,000), MUS81 (mouse monoclonal clone MTA30 2G10/3, sc-53382, Santa Cruz; WB: 1:250), ATM phospho-S1981 (rabbit monoclonal clone EP1890Y, ab81292, Abcam; WB: 1:1,000), ATM (mouse monoclonal clone 1B10, sc-135663, Santa Cruz; WB: 1:100), KAP1 phospho-S824 (rabbit polyclonal, A300-767A, Bethyl Laboratories; WB: 1:1,000), KAP1 (rabbit polyclonal, A300-274A, Bethyl Laboratories; WB: 1:1,000), Chk2 phospho-T68 (rabbit polyclonal, 2661, Cell Signaling Technologies; WB: 1:500), Chk2 (mouse monoclonal clone 8F12, MA5-31595, Invitrogen; WB: 1:500; rabbit monoclonal clone EPR4325, ab109413, Abcam, WB: 1:1,000), H2AX phospho-S139 (rabbit polyclonal, 2577, Cell Signaling Technologies; WB: 1:500; mouse monoclonal clone JBW301, 05-636, Merck, IF: 1:500), Chk1 phospho-S345 (rabbit polyclonal, 2348, Cell Signaling Technologies; WB: 1:1,000), Vinculin (mouse monoclonal clone hVIN-1, V9131, Merck; WB: 1:10,000), EME1 (mouse monoclonal clone MTA31 7h2/1, sc-53275, Santa Cruz; WB: 1:100), TOP2A (mouse monoclonal clone G-6, sc-166934, Santa Cruz; WB: 1:500), UBC9 (goat polyclonal, ab21193, Abcam; WB: 1:500), 53BP1 (rabbit polyclonal, NB100-304, Novus Biologicals; IF: 1:500), NBS1 (rabbit polyclonal, sc-11431, Santa Cruz; WB: 1:1000), SLX4 (rabbit polyclonal, ab100997, Abcam, WB: 1:1,000), phospho-Ser/Thr-Pro MPM-2 Cy5 conjugate (mouse monoclonal clone MPM-2, 16-220, Merck, IF: 1:500), Goat anti-Guinea Pig IgG Alexa Fluor 488 (A-11073, Invitrogen). The following custom-made antibodies were used: NIP45 (sheep polyclonal, raised against full-length human NIP45; WB: 1:1,000; IF: 1:1,000), RMI1 (mouse monoclonal clone TRR-56-8-3; WB: 1:1,000), PICH (guineapig polyclonal; WB: 1:200; IF: 1:500), SLX4 (rabbit polyclonal, gift from John Rouse, University of Dundee; WB: 1:1,000).

Validation

The specificity of antibodies against BLM, EME1, FKBP8, GFP, MUS81, NBS1, NIP45, P300, PICH, RMI1, RMI2, SUMO2/3 and SLX4 were validated using appropriate knockdown/knockout controls in this study (as shown in the manuscript). Other antibodies were used based on previous validation in the literature and/or manufacturer websites: Actin (Lessard, Cell Motil Cytoskeleton 10: 349-362 (1988)); Histone H3 (Abcam); ATM pS1981 (Abcam); ATM (Menotta et al., J Biol Chem 287: 41352–41363 (2012)); KAP1 pS824 (Bethyl Laboratories); KAP1 (Bethyl Laboratories); Chk2 pT68 (Cell Signaling Technologies); rabbit monoclonal Chk2 (Abcam); rabbit polyclonal H2AX pS193 (Cell Signaling Technologies); mouse monoclonal H2AX pS193 (Merck); Chk1 pS345 (Cell Signaling Technologies); Vinculin (Merck); TOP2A (Gothe et al., Mol Cell 75: 267-283.e12 (2019)); 53BP1 (Zelensky et al., PLoS Genet 16: e1008550 (2020)); pS/T MPM-2 (Davis et al., PNAS 80: 2926-2930 (1983)). The following antibodies were not validated to our knowledge: GAPDH, mouse monoclonal Chk2, UBC9.

Eukaryotic cell lines

Policy information about cell lines and Sex and Gender in Research

Cell line source(s)

HeLa cells were obtained from ATCC. RPE1-hTERT PuroS cells were a kind gift from Andrew J. Holland. RPE1-hTERT BLM-KO and parental control cell line were kind gifts from Andrew Blackford. RPE1-hTERT p53-KO FLAG-Cas9 cells were a kid gift from Daniel Durocher. U2OS Flp-In T-REx cells were a kind gift from Helen Piwnica-Worms. U2OS Flp-In T-REx cells inducibly expressing GFP-SLX4 and parental control cells were a kind gift from John Rouse. Generation and validation of HeLa, RPE1-hTERT PuroS, RPE-hTERT p53-KO FLAG-Cas9 and U2OS Flp-In T-REx cell lines with targeted NIP45 KO or RIM1 KO are described in this study.

Authentication

Cell lines were not authenticated.

Mycoplasma contamination

All cell lines used in this study were regularly tested negative for mycoplasma infection.

Commonly misidentified lines
(See ICLAC register)

Cell lines used in this study are not included in the ICLAC register of commonly misidentified cell lines.

Flow Cytometry

Plots

Confirm that:

☒ The axis labels state the marker and fluorochrome used (e.g. CD4-FITC).

☒ The axis scales are clearly visible. Include numbers along axes only for bottom left plot of group (a 'group' is an analysis of identical markers).

☒ All plots are contour plots with outliers or pseudocolor plots.

☒ A numerical value for number of cells or percentage (with statistics) is provided.

Methodology

Sample preparation	Asynchronously growing HeLa wt and NIP45-KO cells were treated or not with IR (4 Gy) followed by nocodazole (150 ng/mL) for 4 h. Cells were collected and fixed in 70% ethanol at 4 °C and stained with phospho-MPM2 antibody (1:1.000) for 2 h at RT.
Instrument	Data was acquired using a 5 laser Becton Dickinson LSR Fortessa (488 nm, 561 nm, 355 nm, 405 nm and 640 nm).
Software	BD FACS Diva software (version 9.0) on the LSR Fortessa was used for data acquisition, and post-acquisition analysis was performed using FCS Express (version 7; DeNovo Software).
Cell population abundance	The cells used in this experiment were only analyzed and not sorted.
Gating strategy	A general gate was created around a population with similar forward and side scatter characteristics. Doublets and aggregates were then excluded using the propidium iodide pulse width measurement. Positive and negative populations were determined using staining controls.

☒ Tick this box to confirm that a figure exemplifying the gating strategy is provided in the Supplementary Information.
