# Supplementary figures and images for: The SUMO–NIP45 pathway processes toxic DNA catenanes to prevent mitotic failure
Source: Nat Struct Mol Biol. 2023 Jul 20;30(9):1303–13. doi: 10.1038/s41594-023-01045-0 (PMC10497417; doi:10.1038/s41594-023-01045-0)

Source Data Fig 1 - Uncropped scans

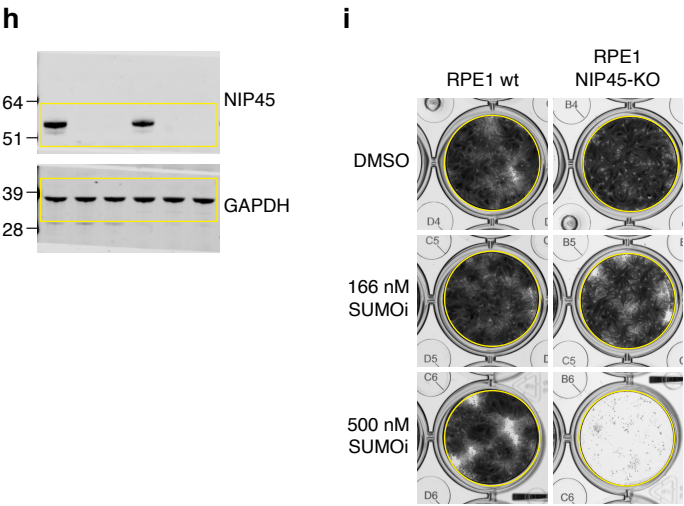

Supplement: Source Data Fig. 1 — Unprocessed western blots and scans. [file 41594_2023_1045_MOESM6_ESM.pdf]

Source Data Fig 2 - Uncropped scans

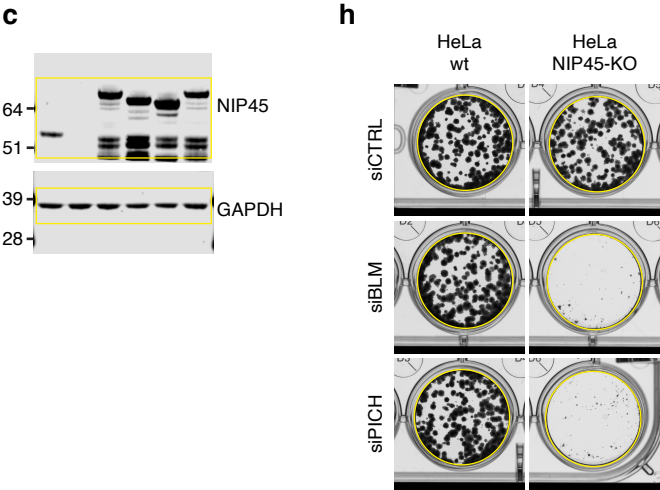

Supplement: Source Data Fig. 2 — Unprocessed western blots and scans. [file 41594_2023_1045_MOESM8_ESM.pdf]

Source Data Fig 5 - Uncropped scans

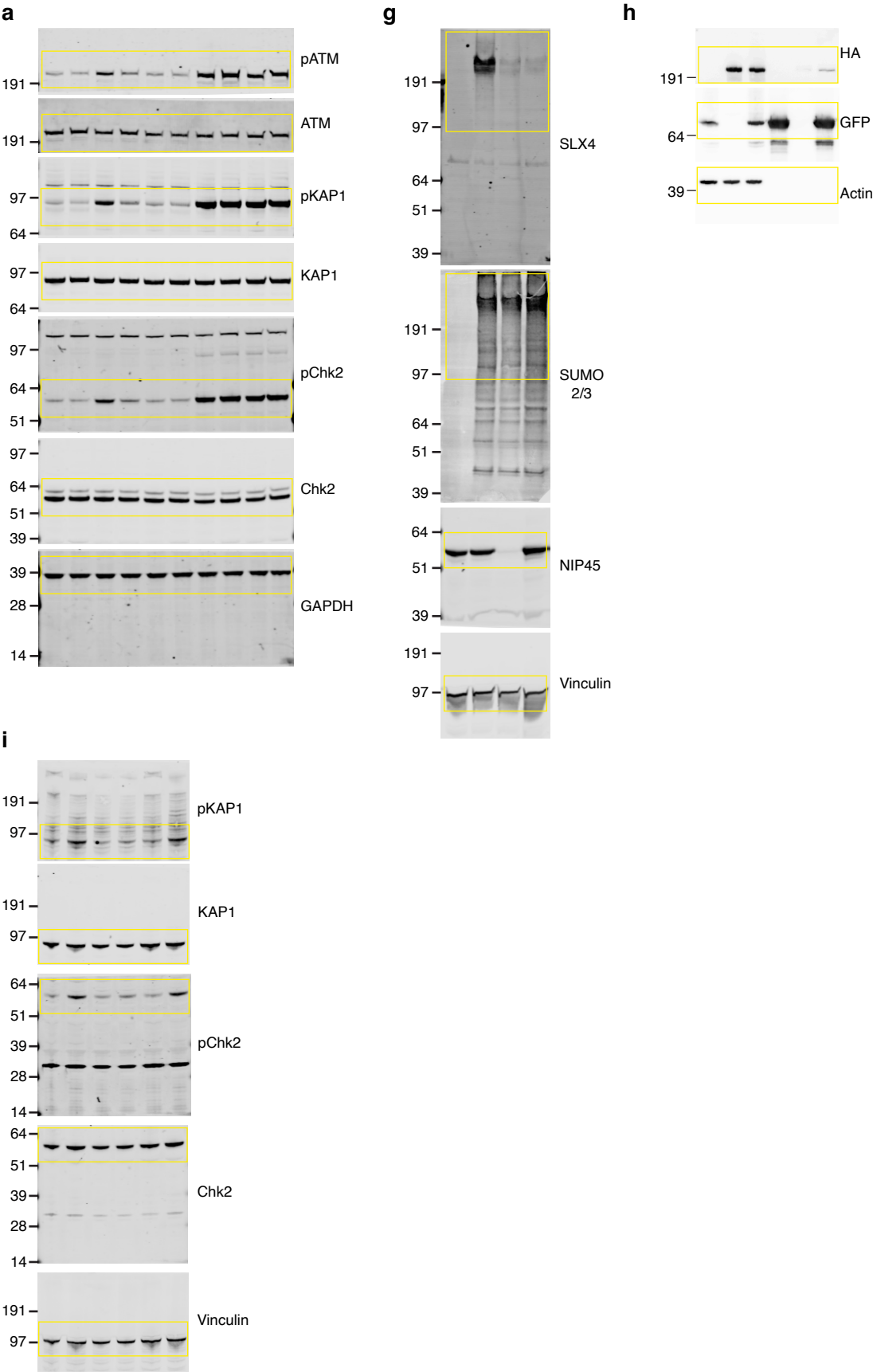

Supplement: Source Data Fig. 5 — Unprocessed western blots. [file 41594_2023_1045_MOESM12_ESM.pdf]

# Source Data Extended Data Fig 1 - Uncropped scans

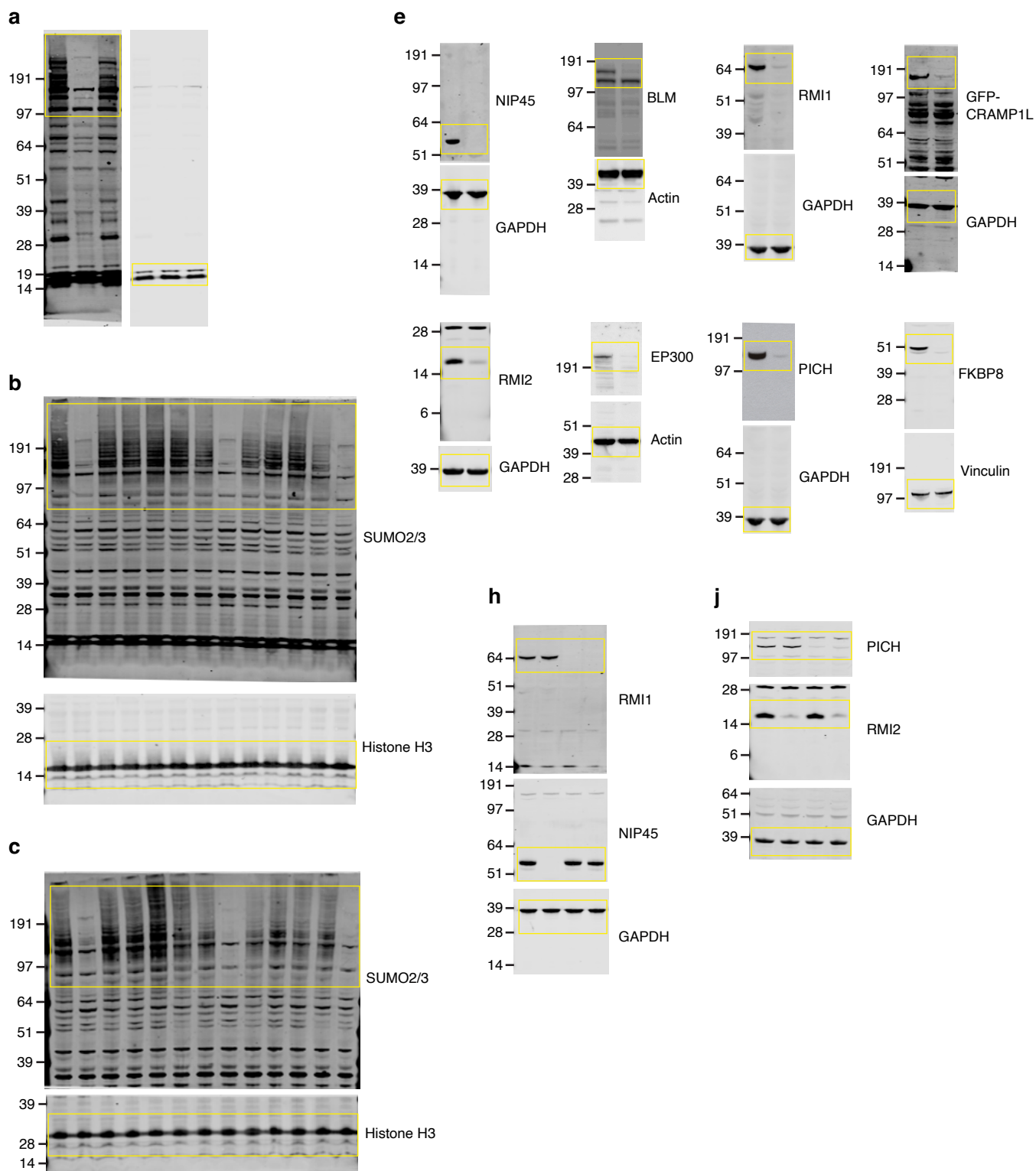

Supplement: Source Data Extended Data Fig. 1 — Unprocessed western blots. [file 41594_2023_1045_MOESM14_ESM.pdf]

Source Data Extended Data Fig 2 - Uncropped scans

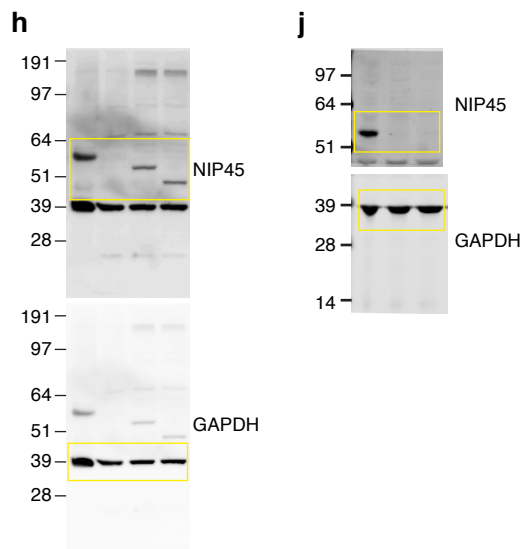

Supplement: Source Data Extended Data Fig. 2 — Unprocessed western blots. [file 41594_2023_1045_MOESM16_ESM.pdf]

Source Data Extended Data Fig 4 - Uncropped scans

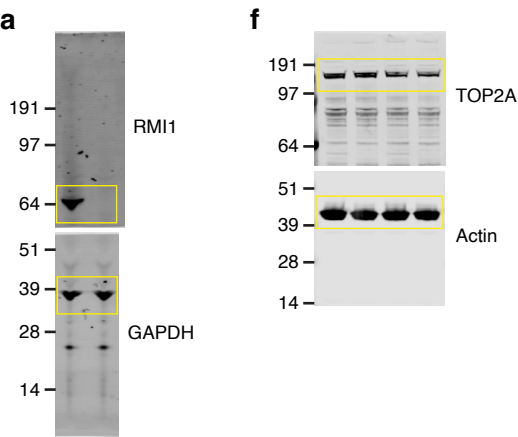

Supplement: Source Data Extended Data Fig. 4 — Unprocessed western blots. [file 41594_2023_1045_MOESM19_ESM.pdf]

Source Data Extended Data Fig 5 - Uncropped scans

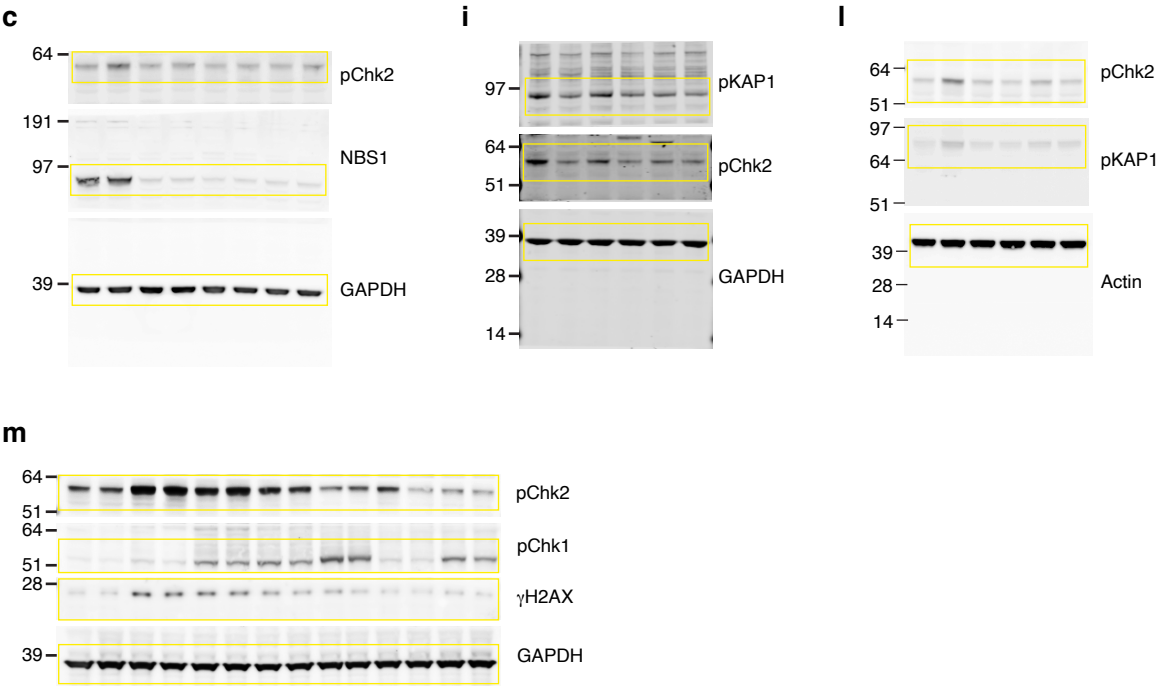

Supplement: Source Data Extended Data Fig. 5 — Unprocessed western blots. [file 41594_2023_1045_MOESM21_ESM.pdf]

Source Data Extended Data Fig 6 - Uncropped scans

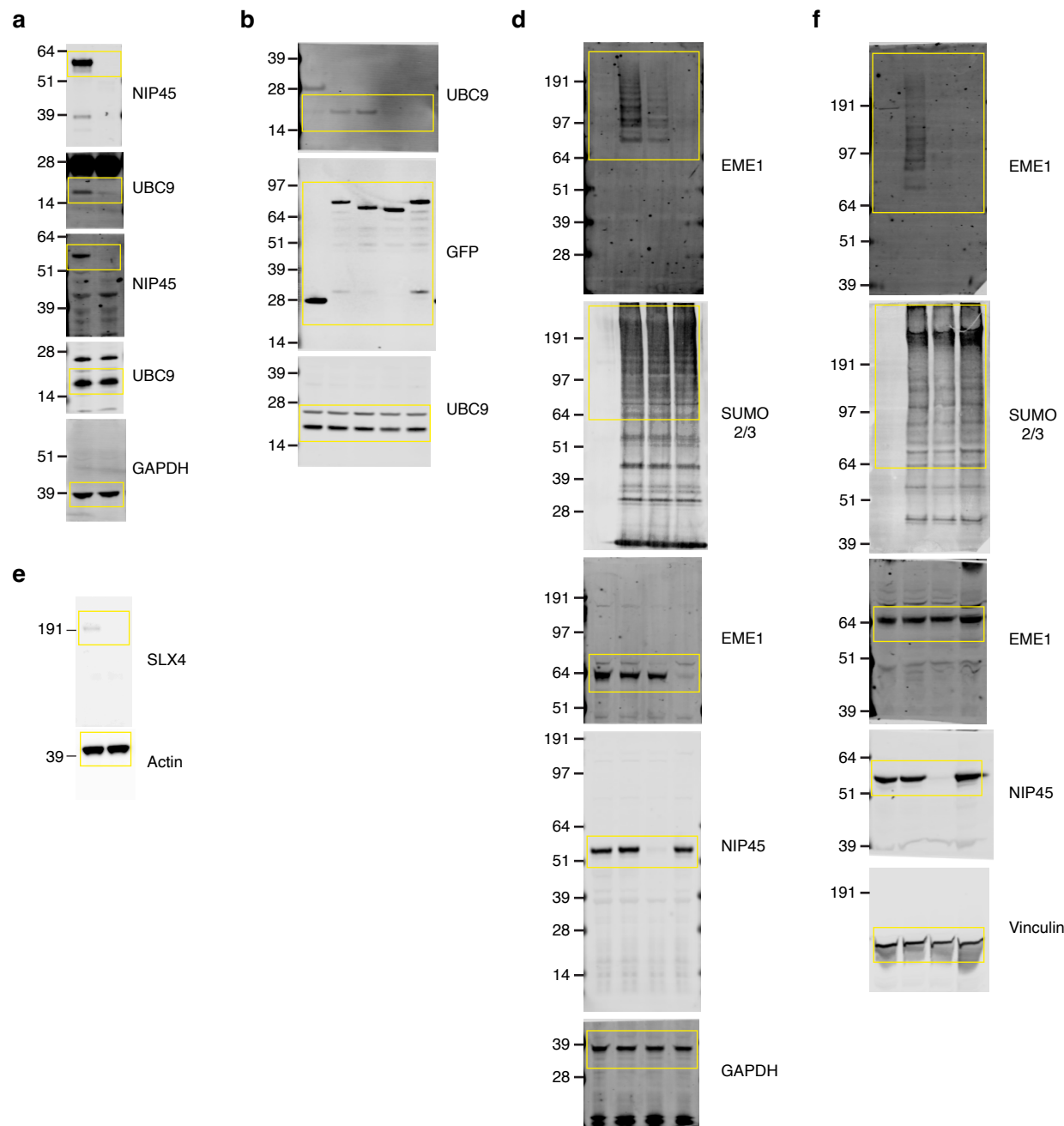

Supplement: Source Data Extended Data Fig. 6 — Unprocessed western blots. [file 41594_2023_1045_MOESM23_ESM.pdf]

Source Data Extended Data Fig 7 - Uncropped scans

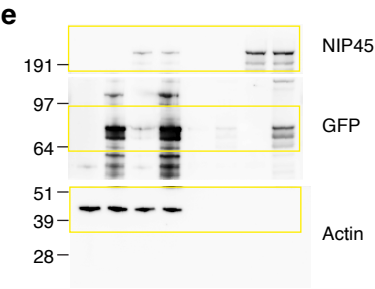

Supplement: Source Data Extended Data Fig. 7 — Unprocessed western blots. [file 41594_2023_1045_MOESM24_ESM.pdf]
